# Poly[hexa-μ-acetato-bis­(dimethyl sulfoxide)­trimanganese(II)]

**DOI:** 10.1107/S1600536810042558

**Published:** 2010-10-30

**Authors:** Chong-Qing Wan, Nai-You Xiao, Zi-Jia Wang

**Affiliations:** a Department of Chemistry, Capital Normal University, Beijing 100048, People’s Republic of China; b China Clean Coal Technology, China Coal Research Institute, Beijing 100013, People’s Republic of China

## Abstract

In the title complex, [Mn_3_(CH_3_CO_2_)_6_(C_2_H_6_SO)_2_]_*n*_, the Mn^II^ ions exhibit similar MnO_6_ octa­hedral coordination geometries but with different coordination environments. One type of Mn^II^ ion is surrounded by five acetate groups and a terminal dimethyl sulfoxide group, while the other lies on a twofold axis and is coordinated by six O atoms from three symmetry-related acetate ions. The acetate anions exhibit three independent bridging modes, which flexibly bridge the Mn^II^ ions along the *c*-axis direction, forming an infinite chain structure; the chains are further inter­connected through weak C—H⋯O and C—H⋯S hydrogen-bonding inter­actions.

## Related literature

For metal complexes of DMSO, see: Calligaris *et al.* (2004 ▶). For the structure of a related complex, see: Wang *et al.* (2000 ▶).
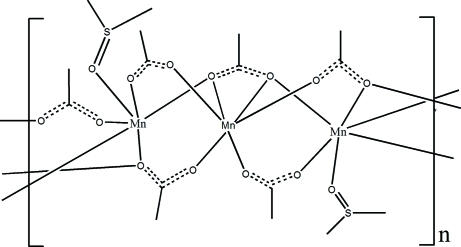


## Experimental

### 

#### Crystal data

[Mn_3_(C_2_H_3_O_2_)_6_(C_2_H_6_OS)_2_]
*M* *_r_* = 675.34
Monoclinic, 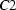

*a* = 12.8475 (16) Å
*b* = 12.5439 (16) Å
*c* = 8.6095 (11) Å
β = 94.906 (2)°
*V* = 1382.4 (3) Å^3^
*Z* = 2
Mo *K*α radiation
μ = 1.56 mm^−1^
*T* = 293 K
0.41 × 0.36 × 0.29 mm

#### Data collection

Bruker SMART CCD area-detector diffractometer
Absorption correction: multi-scan (*SADABS*; Bruker, 2001 ▶) *T*_min_ = 0.883, *T*_max_ = 1.000
3821 measured reflections
1953 independent reflections
1919 reflections with *I* > 2σ(*I*)
*R*_int_ = 0.020

#### Refinement

*R*[*F* ^2^ > 2σ(*F* ^2^)] = 0.021
*wR*(*F* ^2^) = 0.056
*S* = 1.05
1953 reflections
161 parameters
1 restraint
H-atom parameters constrained
Δρ_max_ = 0.39 e Å^−3^
Δρ_min_ = −0.16 e Å^−3^
Absolute structure: Flack (1983 ▶), 653 Friedel pairs
Flack parameter: 0.034 (17)

### 

Data collection: *APEX2* (Bruker, 2007 ▶); cell refinement: *SAINT* (Bruker, 2007 ▶); data reduction: *SAINT*; program(s) used to solve structure: *SHELXS97* (Sheldrick, 2008 ▶); program(s) used to refine structure: *SHELXL97* (Sheldrick, 2008 ▶); molecular graphics: *SHELXTL* (Sheldrick, 2008 ▶); software used to prepare material for publication: *SHELXTL* and *PLATON* (Spek, 2009 ▶).

## Supplementary Material

Crystal structure: contains datablocks I, global. DOI: 10.1107/S1600536810042558/pv2332sup1.cif

Structure factors: contains datablocks I. DOI: 10.1107/S1600536810042558/pv2332Isup2.hkl

Additional supplementary materials:  crystallographic information; 3D view; checkCIF report

## Figures and Tables

**Table 1:** Hydrogen-bond geometry (Å, °)

*D*—H⋯*A*	*D*—H	H⋯*A*	*D*⋯*A*	*D*—H⋯*A*
C8—H8*B*⋯O6^i^	0.96	2.45	3.367 (4)	160
C2—H2*B*⋯S1^ii^	0.96	2.99	3.841 (4)	147

Symmetry codes: (i) 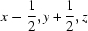; (ii) 
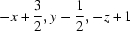
.

**Fig. 1.**
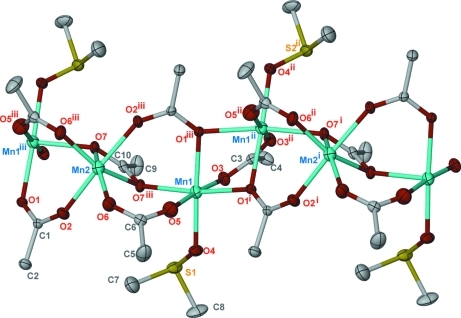
A view of the title complex with the atom-numbering scheme; hydrogen atoms are omitted for clarity. Displacement ellipsoids are drawn at 30% probability level. Symmetry codes: i x, y, z-1; ii -x+2,y,-z; iii -x + 2, y, -z +1.

**Fig. 2.**
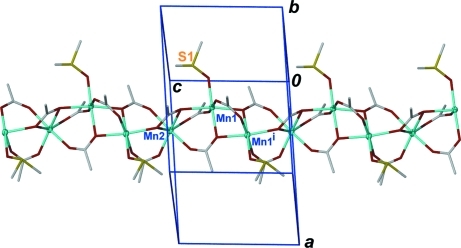
Infinite chain of the MnII ions bridged by acetate anions along the c direction in a unit cell. Symmetry code: i -x + 2, y, -z.

## supplementary crystallographic information

### Comment

The coordination chemistry of dimethyl sulfoxid (DMSO) has been widely studied. Herein, we report the preparation and crystal strcuture of a new manganese(II) complex with dimethyl sulfoxide (DMSO). In the title complex, the two independent Mn^II^ ions (Mn1 and Mn2) exhibit a similar *O6*-octahedral coordination geometry with different coordination environments (Fig. 1). The Mn1 ion is surrounded by five acetates and one η^1^-bonding DMSO, while the Mn2 lies on a two-fold axis and is coordinated by six oxygen atoms of three symmetry related acetate ions. The acetate anions exhibit three independent bridging modes, *syn*, *syn*η^1^:η^1^:µ^2^-mode (C2-symmetric O3-containing acetate and O5-, O6-containing acetate), the *syn*, *syn*, *ant*η^1^:η^2^:µ^3^-mode (O1-, O2-containing acetate) and the *syn*, *ant*, *syn*, *ant*η^2^:η^2^:µ^3^-mode (C2-symmetric O7-containing acetate). The Mn1 and Mn2 ions are flexibly bridged by these anions and assemble into an infinite chain along the *c* direction (Fig. 2). The parallel arrays interconnect through C—H···O and C—H···S type H-bonding interactions (Table 1). In the termianl dimethyl sulfoxide, the S1═O4 of 1.501 (2)Å bond is slightly longer than that of the neat DMSO, which can be ascribed to the reduced bond order as that found in the protonated and η^1^-coordinated alkyl sulfoxides (Calligaris *et al.*, 2004). The Mn1—O4 bond length of 2.153 (2)Å is comparable to 2.158 (2)Å found in catena-(tetrakis(µ_2_-thiocyanato-N,S)-bis(dimethyl sulfoxide-O)- manganese(II)-mercury(II) (Wang *et al.*, 2000), in which the dimethyl sulfoxide shows a similar terminal η^1^-coordinated bonding to the Mn^II^.

### Experimental

Mn(CH_3_CO_2_)_2_.4H_2_O (25 mg, 0.1 mmol) was dissolved in 3 ml deionized water with stirring at room temperature. After half an hour, 1 ml dimethyl sulfoxide was added to the solution. The mixed solution was stirred for another half hour, and then filtered. The clear solution obtained was left to stand in the air to let the solvent to evaporate. The colorless crystals were deposited after one week (12.60 mg, yield 56%).

### Refinement

An absolute structure was determined using the Flack (1983) method. The hydrogen atoms were placed in idealized positions and allowed to ride on the parent carbon atoms, with C—H = 0.96 Å and *U_iso_*(H) = 1.5*U_eq_*(C).

### Crystal data

**Table:**

[Mn_3_(C_2_H_3_O_2_)_6_(C_2_H_6_OS)_2_]	*Z* = 2
*M**_r_* = 675.34	*F*(000) = 690
Monoclinic, *C*2	*D*_x_ = 1.622 Mg m^−^^3^
Hall symbol: C 2y	Mo *K*α radiation, λ = 0.71073 Å
*a* = 12.8475 (16) Å	θ = 2.4–25.1°
*b* = 12.5439 (16) Å	µ = 1.56 mm^−^^1^
*c* = 8.6095 (11) Å	*T* = 293 K
β = 94.906 (2)°	Block, colorless
*V* = 1382.4 (3) Å^3^	0.41 × 0.36 × 0.29 mm

### Data collection

**Table:**

Bruker SMART CCD area-detector diffractometer	1953 independent reflections
Radiation source: fine-focus sealed tube	1919 reflections with *I* > 2σ(*I*)
graphite	*R*_int_ = 0.020
ω scans	θ_max_ = 25.1°, θ_min_ = 2.4°
Absorption correction: multi-scan (*SADABS*; Bruker, 2001)	*h* = −15→15
*T*_min_ = 0.883, *T*_max_ = 1.000	*k* = −12→14
3821 measured reflections	*l* = −10→9

### Refinement

**Table:**

Refinement on *F*^2^	Secondary atom site location: difference Fourier map
Least-squares matrix: full	Hydrogen site location: inferred from neighbouring sites
*R*[*F*^2^ > 2σ(*F*^2^)] = 0.021	H-atom parameters constrained
*wR*(*F*^2^) = 0.056	*w* = 1/[σ^2^(*F*_o_^2^) + (0.033*P*)^2^ + 0.3155*P*] *P* = (*F*_o_^2^ + 2*F*_c_^2^)/3
*S* = 1.05	(Δ/σ)_max_ = 0.001
1953 reflections	Δρ_max_ = 0.39 e Å^−^^3^
161 parameters	Δρ_min_ = −0.16 e Å^−^^3^
1 restraint	Absolute structure: Flack (1983), 653 Friedel pairs
Primary atom site location: structure-invariant direct methods	Flack parameter: 0.034 (17)

### Special details

**Table:**

Geometry. All esds (except the esd in the dihedral angle between two l.s. planes) are estimated using the full covariance matrix. The cell esds are taken into account individually in the estimation of esds in distances, angles and torsion angles; correlations between esds in cell parameters are only used when they are defined by crystal symmetry. An approximate (isotropic) treatment of cell esds is used for estimating esds involving l.s. planes.
Refinement. Refinement of F^2^ against ALL reflections. The weighted R-factor wR and goodness of fit S are based on F^2^, conventional R-factors R are based on F, with F set to zero for negative F^2^. The threshold expression of F^2^ > 2sigma(F^2^) is used only for calculating R-factors(gt) etc. and is not relevant to the choice of reflections for refinement. R-factors based on F^2^ are statistically about twice as large as those based on F, and R- factors based on ALL data will be even larger. An absolute structure was established with the Flack parameter of 0.034 (17).

### Fractional atomic coordinates and isotropic or equivalent isotropic displacement parameters (Å^2^)

**Table:**

	*x*	*y*	*z*	*U*_iso_*/*U*_eq_	Occ. (<1)
Mn1	0.91731 (2)	0.56819 (3)	0.13931 (4)	0.02995 (11)	
Mn2	1.0000	0.43103 (4)	0.5000	0.03045 (14)	
S1	0.70961 (5)	0.67013 (7)	0.27681 (10)	0.0504 (2)	
O1	0.91650 (12)	0.53278 (16)	0.88461 (19)	0.0352 (4)	
O2	0.87911 (14)	0.44946 (19)	0.6590 (2)	0.0447 (5)	
O3	0.94917 (16)	0.73193 (16)	0.0982 (2)	0.0446 (5)	
O4	0.75455 (15)	0.6046 (2)	0.1528 (2)	0.0526 (6)	
O5	0.87347 (19)	0.4057 (2)	0.1696 (3)	0.0641 (6)	
O6	0.89783 (16)	0.3166 (2)	0.3901 (2)	0.0517 (5)	
O7	1.05675 (14)	0.59438 (15)	0.60213 (19)	0.0379 (4)	
C1	0.85462 (19)	0.4827 (2)	0.7865 (3)	0.0318 (5)	
C2	0.7442 (2)	0.4630 (3)	0.8279 (4)	0.0454 (7)	
H2A	0.7355	0.4928	0.9287	0.068*	
H2B	0.7312	0.3877	0.8299	0.068*	
H2C	0.6958	0.4961	0.7515	0.068*	
C3	1.0000	0.7770 (3)	0.0000	0.0376 (8)	
C4	1.0000	0.8965 (4)	0.0000	0.0634 (14)	
H4A	1.0425	0.9220	−0.0786	0.095*	0.50
H4B	1.0277	0.9220	0.1003	0.095*	0.50
H4C	0.9298	0.9220	−0.0217	0.095*	0.50
C5	0.7587 (3)	0.2664 (4)	0.2103 (5)	0.0792 (13)	
H5A	0.7291	0.2898	0.1099	0.119*	
H5B	0.7069	0.2712	0.2841	0.119*	
H5C	0.7814	0.1937	0.2033	0.119*	
C6	0.8501 (2)	0.3355 (2)	0.2627 (3)	0.0386 (6)	
C7	0.6698 (3)	0.5766 (5)	0.4144 (5)	0.0873 (14)	
H7A	0.7303	0.5483	0.4736	0.131*	
H7B	0.6320	0.5197	0.3607	0.131*	
H7C	0.6257	0.6113	0.4834	0.131*	
C8	0.5846 (3)	0.7060 (4)	0.1907 (5)	0.0799 (13)	
H8A	0.5918	0.7598	0.1130	0.120*	
H8B	0.5432	0.7333	0.2694	0.120*	
H8C	0.5511	0.6444	0.1429	0.120*	
C9	1.0000	0.7628 (4)	0.5000	0.0727 (16)	
H9A	0.9531	0.7883	0.4154	0.109*	0.50
H9B	1.0693	0.7883	0.4878	0.109*	0.50
H9C	0.9776	0.7883	0.5969	0.109*	0.50
C10	1.0000	0.6450 (4)	0.5000	0.0383 (9)	

### Atomic displacement parameters (Å^2^)

**Table:**

	*U*^11^	*U*^22^	*U*^33^	*U*^12^	*U*^13^	*U*^23^
Mn1	0.02796 (17)	0.0394 (2)	0.02291 (18)	0.00325 (16)	0.00453 (12)	0.00263 (16)
Mn2	0.0318 (3)	0.0360 (3)	0.0238 (3)	0.000	0.00371 (19)	0.000
S1	0.0378 (4)	0.0585 (5)	0.0547 (5)	0.0060 (3)	0.0025 (3)	−0.0202 (4)
O1	0.0278 (8)	0.0502 (11)	0.0278 (9)	−0.0062 (8)	0.0037 (7)	−0.0038 (8)
O2	0.0401 (9)	0.0648 (14)	0.0305 (9)	−0.0086 (9)	0.0107 (8)	−0.0127 (9)
O3	0.0549 (11)	0.0398 (11)	0.0407 (11)	0.0029 (9)	0.0141 (9)	0.0029 (8)
O4	0.0341 (10)	0.0810 (17)	0.0428 (11)	0.0108 (9)	0.0043 (8)	−0.0150 (11)
O5	0.0691 (15)	0.0525 (15)	0.0704 (16)	−0.0129 (12)	0.0037 (12)	0.0102 (12)
O6	0.0554 (11)	0.0611 (14)	0.0382 (11)	−0.0123 (10)	0.0010 (9)	−0.0110 (10)
O7	0.0454 (9)	0.0452 (12)	0.0229 (8)	−0.0039 (8)	0.0019 (7)	0.0009 (8)
C1	0.0285 (12)	0.0419 (14)	0.0248 (12)	−0.0027 (10)	0.0020 (10)	0.0013 (11)
C2	0.0345 (13)	0.065 (2)	0.0369 (15)	−0.0113 (13)	0.0062 (11)	−0.0079 (13)
C3	0.0337 (17)	0.039 (2)	0.040 (2)	0.000	−0.0017 (15)	0.000
C4	0.061 (3)	0.041 (2)	0.092 (4)	0.000	0.031 (3)	0.000
C5	0.078 (2)	0.087 (3)	0.068 (2)	−0.039 (2)	−0.020 (2)	0.009 (2)
C6	0.0384 (13)	0.0347 (14)	0.0433 (15)	−0.0019 (11)	0.0079 (11)	−0.0041 (12)
C7	0.076 (2)	0.134 (4)	0.054 (2)	−0.005 (3)	0.0194 (18)	0.000 (3)
C8	0.0454 (17)	0.081 (3)	0.110 (3)	0.0282 (19)	−0.0133 (19)	−0.029 (3)
C9	0.120 (5)	0.046 (3)	0.051 (3)	0.000	0.000 (3)	0.000
C10	0.047 (2)	0.043 (2)	0.0272 (19)	0.000	0.0116 (17)	0.000

### Geometric parameters (Å, °)

**Table:**

Mn1—O3	2.130 (2)	C1—C2	1.513 (4)
Mn1—O5	2.136 (2)	C2—H2A	0.9600
Mn1—O4	2.1533 (19)	C2—H2B	0.9600
Mn1—O1^i^	2.2076 (15)	C2—H2C	0.9600
Mn1—O1^ii^	2.2365 (17)	C3—O3^iv^	1.247 (3)
Mn1—O7^i^	2.2467 (17)	C3—C4	1.498 (6)
Mn2—O6	2.113 (2)	C4—H4A	0.9600
Mn2—O6^i^	2.113 (2)	C4—H4B	0.9600
Mn2—O2	2.1690 (18)	C4—H4C	0.9600
Mn2—O2^i^	2.1690 (18)	C5—C6	1.499 (5)
Mn2—O7	2.3224 (19)	C5—H5A	0.9600
Mn2—O7^i^	2.3224 (19)	C5—H5B	0.9600
S1—O4	1.501 (2)	C5—H5C	0.9600
S1—C8	1.768 (3)	C7—H7A	0.9600
S1—C7	1.773 (5)	C7—H7B	0.9600
O1—C1	1.275 (3)	C7—H7C	0.9600
O1—Mn1^i^	2.2076 (15)	C8—H8A	0.9600
O1—Mn1^iii^	2.2365 (17)	C8—H8B	0.9600
O2—C1	1.239 (3)	C8—H8C	0.9600
O3—C3	1.247 (3)	C9—C10	1.477 (7)
O5—C6	1.245 (4)	C9—H9A	0.9600
O6—C6	1.233 (4)	C9—H9B	0.9600
O7—C10	1.264 (3)	C9—H9C	0.9600
O7—Mn1^i^	2.2467 (17)	C10—O7^i^	1.264 (3)
			
O3—Mn1—O5	175.34 (9)	O1—C1—C2	117.9 (2)
O3—Mn1—O4	90.32 (9)	C1—C2—H2A	109.5
O5—Mn1—O4	85.91 (10)	C1—C2—H2B	109.5
O3—Mn1—O1^i^	88.70 (8)	H2A—C2—H2B	109.5
O5—Mn1—O1^i^	94.97 (9)	C1—C2—H2C	109.5
O4—Mn1—O1^i^	177.66 (7)	H2A—C2—H2C	109.5
O3—Mn1—O1^ii^	90.78 (7)	H2B—C2—H2C	109.5
O5—Mn1—O1^ii^	87.19 (9)	O3^iv^—C3—O3	126.1 (4)
O4—Mn1—O1^ii^	99.89 (7)	O3^iv^—C3—C4	116.97 (19)
O1^i^—Mn1—O1^ii^	78.00 (7)	O3—C3—C4	116.97 (19)
O3—Mn1—O7^i^	90.53 (7)	C3—C4—H4A	109.5
O5—Mn1—O7^i^	92.12 (9)	C3—C4—H4B	109.5
O4—Mn1—O7^i^	88.74 (7)	H4A—C4—H4B	109.5
O1^i^—Mn1—O7^i^	93.39 (6)	C3—C4—H4C	109.5
O1^ii^—Mn1—O7^i^	171.26 (6)	H4A—C4—H4C	109.5
O6—Mn2—O6^i^	94.44 (13)	H4B—C4—H4C	109.5
O6—Mn2—O2	84.50 (8)	C6—C5—H5A	109.5
O6^i^—Mn2—O2	103.92 (8)	C6—C5—H5B	109.5
O6—Mn2—O2^i^	103.92 (8)	H5A—C5—H5B	109.5
O6^i^—Mn2—O2^i^	84.50 (8)	C6—C5—H5C	109.5
O2—Mn2—O2^i^	167.76 (13)	H5A—C5—H5C	109.5
O6—Mn2—O7	158.69 (8)	H5B—C5—H5C	109.5
O6^i^—Mn2—O7	105.48 (8)	O6—C6—O5	125.5 (3)
O2—Mn2—O7	83.42 (7)	O6—C6—C5	118.3 (3)
O2^i^—Mn2—O7	85.78 (8)	O5—C6—C5	116.2 (3)
O6—Mn2—O7^i^	105.48 (8)	S1—C7—H7A	109.5
O6^i^—Mn2—O7^i^	158.69 (8)	S1—C7—H7B	109.5
O2—Mn2—O7^i^	85.78 (8)	H7A—C7—H7B	109.5
O2^i^—Mn2—O7^i^	83.42 (7)	S1—C7—H7C	109.5
O7—Mn2—O7^i^	56.16 (9)	H7A—C7—H7C	109.5
O4—S1—C8	103.40 (16)	H7B—C7—H7C	109.5
O4—S1—C7	105.3 (2)	S1—C8—H8A	109.5
C8—S1—C7	98.3 (2)	S1—C8—H8B	109.5
C1—O1—Mn1^i^	126.19 (15)	H8A—C8—H8B	109.5
C1—O1—Mn1^iii^	134.27 (15)	S1—C8—H8C	109.5
Mn1^i^—O1—Mn1^iii^	97.36 (6)	H8A—C8—H8C	109.5
C1—O2—Mn2	147.63 (17)	H8B—C8—H8C	109.5
C3—O3—Mn1	132.0 (2)	C10—C9—H9A	109.5
S1—O4—Mn1	126.07 (12)	C10—C9—H9B	109.5
C6—O5—Mn1	146.5 (2)	H9A—C9—H9B	109.5
C6—O6—Mn2	120.7 (2)	C10—C9—H9C	109.5
C10—O7—Mn1^i^	142.22 (15)	H9A—C9—H9C	109.5
C10—O7—Mn2	92.1 (2)	H9B—C9—H9C	109.5
Mn1^i^—O7—Mn2	105.15 (7)	O7^i^—C10—O7	119.7 (4)
O2—C1—O1	124.1 (2)	O7^i^—C10—C9	120.16 (19)
O2—C1—C2	117.9 (2)	O7—C10—C9	120.16 (19)
			
O6—Mn2—O2—C1	163.4 (4)	O6—Mn2—O7—C10	−33.4 (2)
O6^i^—Mn2—O2—C1	70.2 (4)	O6^i^—Mn2—O7—C10	168.01 (9)
O2^i^—Mn2—O2—C1	−62.4 (4)	O2—Mn2—O7—C10	−89.32 (10)
O7—Mn2—O2—C1	−34.2 (4)	O2^i^—Mn2—O7—C10	84.91 (9)
O7^i^—Mn2—O2—C1	−90.5 (4)	O7^i^—Mn2—O7—C10	0.0
O5—Mn1—O3—C3	−96.9 (11)	O6—Mn2—O7—Mn1^i^	112.59 (19)
O4—Mn1—O3—C3	−132.66 (18)	O6^i^—Mn2—O7—Mn1^i^	−45.97 (9)
O1^i^—Mn1—O3—C3	45.22 (18)	O2—Mn2—O7—Mn1^i^	56.70 (8)
O1^ii^—Mn1—O3—C3	−32.76 (18)	O2^i^—Mn2—O7—Mn1^i^	−129.08 (8)
O7^i^—Mn1—O3—C3	138.60 (19)	O7^i^—Mn2—O7—Mn1^i^	146.02 (13)
C8—S1—O4—Mn1	161.6 (2)	Mn2—O2—C1—O1	2.3 (6)
C7—S1—O4—Mn1	−95.7 (2)	Mn2—O2—C1—C2	−177.9 (3)
O3—Mn1—O4—S1	−66.55 (18)	Mn1^i^—O1—C1—O2	−2.6 (4)
O5—Mn1—O4—S1	116.18 (19)	Mn1^iii^—O1—C1—O2	−161.7 (2)
O1^i^—Mn1—O4—S1	−132 (2)	Mn1^i^—O1—C1—C2	177.7 (2)
O1^ii^—Mn1—O4—S1	−157.39 (17)	Mn1^iii^—O1—C1—C2	18.5 (4)
O7^i^—Mn1—O4—S1	23.97 (18)	Mn1—O3—C3—O3^iv^	−3.74 (11)
O3—Mn1—O5—C6	−114.0 (11)	Mn1—O3—C3—C4	176.26 (11)
O4—Mn1—O5—C6	−78.1 (4)	Mn2—O6—C6—O5	18.9 (4)
O1^i^—Mn1—O5—C6	104.1 (4)	Mn2—O6—C6—C5	−163.8 (3)
O1^ii^—Mn1—O5—C6	−178.2 (4)	Mn1—O5—C6—O6	−46.7 (6)
O7^i^—Mn1—O5—C6	10.5 (4)	Mn1—O5—C6—C5	135.9 (4)
O6^i^—Mn2—O6—C6	−148.6 (2)	Mn1^i^—O7—C10—O7^i^	−118.3 (3)
O2—Mn2—O6—C6	107.8 (2)	Mn2—O7—C10—O7^i^	0.0
O2^i^—Mn2—O6—C6	−63.2 (2)	Mn1^i^—O7—C10—C9	61.7 (3)
O7—Mn2—O6—C6	52.1 (3)	Mn2—O7—C10—C9	180.0
O7^i^—Mn2—O6—C6	23.7 (2)		

Symmetry codes: (i) −*x*+2, *y*, −*z*+1; (ii) *x*, *y*, *z*−1; (iii) *x*, *y*, *z*+1; (iv) −*x*+2, *y*, −*z*.

### Hydrogen-bond geometry (Å, °)

**Table:**

*D*—H···*A*	*D*—H	H···*A*	*D*···*A*	*D*—H···*A*
C8—H8B···O6^v^	0.96	2.45	3.367 (4)	160
C2—H2B···S1^vi^	0.96	2.99	3.841 (4)	147

Symmetry codes: (v) *x*−1/2, *y*+1/2, *z*; (vi) −*x*+3/2, *y*−1/2, −*z*+1.

## References

[bb1] Bruker (2001). *SADABS* Bruker AXS Inc., Madison, Wisconsin, USA.

[bb2] Bruker (2007). *APEX2* and *SAINT* Bruker AXS Inc., Madison, Wisconsin, USA.

[bb3] Calligaris, M. (2004). *Coord. Chem. Rev.* **248**, 351–375.

[bb4] Flack, H. D. (1983). *Acta Cryst.* A**39**, 876–881.

[bb5] Sheldrick, G. M. (2008). *Acta Cryst.* A**64**, 112–122. 10.1107/S0108767307043930 18156677

[bb6] Spek, A. L. (2009). *Acta Cryst.* D**65**, 148–155. 10.1107/S090744490804362X PMC2631630 19171970

[bb7] Wang, X. Q., Yu, W. T., Xu, D., Lu, M. K. & Yuan, D. R. (2000). *Acta Cryst.* C**56**, 418–420. 10.1107/s0108270199016765 10815192

